# The Editor's Letter-Box

**Published:** 1913-08-09

**Authors:** Margaret Breay

**Affiliations:** Hon. Secretary.


					Ti ? ? [Copy.]
"e Society for the State Registration of Trained Nurses,
431 Oxford Street, London, W.,
July 25, 1913.
S*?,?I am directed to forward for your information a
^?Py of a resolution adopted at the Annual Meeting of the
'-ociety for the State Registration of Trained Nurses, held
ln London on July 18, which has been sent to the Secretary
^ the London Hospital, desiring that he will bring it
efore the next meeting of the House Committee of the
hospital.?I am, Sir, yours faithfully,
(Signed)
Margaret Breay, Hon. Secretary.

				

